# Decision support for chronic pain care: how do primary care physicians decide when to prescribe opioids? a qualitative study

**DOI:** 10.1186/s12875-015-0264-3

**Published:** 2015-04-14

**Authors:** Christopher A Harle, Sarah E Bauer, Huong Q Hoang, Robert L Cook, Robert W Hurley, Roger B Fillingim

**Affiliations:** Department of Health Services Research, Management and Policy, University of Florida, Gainesville, FL USA; Department of Epidemiology, University of Florida, Gainesville, FL USA; Department of Anesthesiology, Medical College of Wisconsin, Milwaukee, WI USA; Department of Community Dentistry and Behavioral Science, University of Florida, Gainesville, FL USA; Mayo Clinic, Rochester, MN USA

**Keywords:** Clinical decision support, Information needs, Decision making, Chronic pain, Opioids, Primary care, Health care quality

## Abstract

**Background:**

Primary care physicians struggle to treat chronic noncancer pain while limiting opioid misuse, abuse, and diversion. The objective of this study was to understand how primary care physicians perceive their decisions to prescribe opioids in the context of chronic noncancer pain management. This question is important because interventions, such as decision support tools, must be designed based on a detailed understanding of how clinicians use information to make care decisions.

**Methods:**

We conducted in-depth qualitative interviews with family medicine and general internal medicine physicians until reaching saturation in emergent themes. We used a funneling approach to ask a series of questions about physicians’ general decision making challenges and use of information when considering chronic opioids. We then used an iterative, open-coding approach to identify and characterize themes in the data.

**Results:**

We interviewed fifteen physicians with diverse clinical experiences, demographics, and practice affiliations. Physicians said that general decision making challenges in providing pain management included weighing risks and benefits of opioid therapies and time and resource constraints. Also, some physicians described their active avoidance of chronic pain treatment due to concerns about opioid risks. In their decision making, physicians described the importance of objective and consistent information, the importance of identifying “red flags” related to risks of opioids, the importance of information about physical function as an outcome, and the importance of information that engenders trust in patients.

**Conclusions:**

This study identified and described primary care physicians’ struggles to deliver high quality care as they seek and make decisions based on an array of incomplete, conflicting, and often untrusted patient information. Decision support systems, education, and other interventions that address these challenges may alleviate primary care physicians’ struggles and improve outcomes for patients with chronic pain and other challenging conditions.

## Background

Chronic pain imposes a major clinical, social and economic burden, affecting an estimated 100 million Americans and costing $600 billion annually in health care and lost worker productivity [1,2]. Because of the limited number of pain specialist physicians, primary care clinicians provide much of the health care systems’ pain care. Primary care physicians report frustration when caring for patients with chronic pain, much of which relates to concerns about opioid misuse, abuse, and diversion [3-8]. Furthermore, primary care physicians often have minimal training in pain care [9] and practical time constraints [10,11]. As the adoption of health information technology proliferates, well-designed clinical decision support can help primary care physicians efficiently obtain and use the relevant, accurate, and timely information needed to prescribe opioids or alternate pain therapies effectively [12-15].

Prior research has examined physicians’ challenges during encounters involving opioids [3,4,8], patient and physician characteristics related to opioid prescribing [16-18], and physician attitudes toward opioids [19,5,6,8,20]. But, research has not more closely characterized physicians’ use of information and decision processes when deciding whether or not to prescribe opioids. This knowledge is needed so information systems designers, policymakers, and administrators can design usable and useful systems of care. For example, some U.S.-based clinical guidelines [21,22] recommend that physicians access and use specific information, such as opioid risk assessment screeners [23,24], urine drug screening, standardized pain scales, and prescription drug monitoring databases. However, evidence suggests physicians do not widely use these tools [25-28]. As is often the case with systems that suffer from poor adoption, physician’s failure to use guideline-recommended information may stem from uncertain value of the information as well as a practical lack of time and resources [10,11,15]. Thus, there is a need for a detailed understanding of physicians’ information needs and clinical decision making processes. With this understanding, new systems, such as decision support tools, may be better designed with the physicians’ unique work needs in mind. Once implemented, such tools may help clinicians overcome common barriers, such as lack of information or time constraints, to the delivery of guideline-based chronic opioid and chronic noncancer pain management.

The objective of this study was to understand how primary care physicians perceive their decisions to prescribe opioids in the context of chronic noncancer pain management. As described above, this is important because systems interventions, such as decision support tools, must be designed based on an understanding of how clinicians perceive and make decisions in the context of their daily practice. To answer this question, we conducted in-depth qualitative interviews with primary care physicians with varying experience and training in pain care. Interview techniques are commonly used in medical decision making research (cite) and are particularly appropriate for this study because it aims to inform new decision support tools that physicians will be likely to adopt and regularly use in their clinical practice.

## Methods

We conducted in-depth qualitative interviews in the spring of 2013 with family medicine and general internal medicine physicians practicing in the Gainesville and Jacksonville areas of north-central Florida. Participation was voluntary, and all participating physicians gave written informed consent to participate. The interviews were recorded and then transcribed. Identifying information was removed from the transcripts to preserve physicians’ confidentiality. The study was approved by the University of Florida Institutional Review Board.

To obtain a diverse set of perspectives and fully understand physician decision making, we purposefully recruited from nine practices that spanned rural and urban settings and that had different racial, ethnic, and socioeconomic patient mixes. Furthermore, the practices varied in organizational structure, including private practices, United States Veteran’s Health Administration practices, academic practices located on a university campus, and academic-affiliated practices that function as independent community practices. We used emails and phone calls to invite individuals to participate. To further achieve sample diversity, we recruited physicians who varied in terms of age, gender, and experience or training in pain care.

### Procedure

We developed a series of semi-structured interview questions that followed a funneling approach, beginning with broad questions, such as “tell me about your patients,” and then moving to increasingly specific questions, such as “tell me about how you manage patients with chronic non-cancer pain” and “tell me about the decision process you go through when you consider whether to prescribe opioids.” Each question was followed by probing questions to further clarify physicians’ use of information and use of other resources and decision strategies. We also included probing questions that asked about common challenges and information needs. To help understand how physicians’ perceived decisions align with guideline recommendations, a number of the most specific questions asked if and how physicians use strategies that are recommended by clinical practice guidelines for chronic opioid therapy, such as risk assessment and medication tapering or discontinuation [29].

The lead author [CAH] conducted each interview one-on-one. The lead author is a non-clinician health services and health informatics researcher. He had no prior relationship with any of the participants. Each interview lasted 30-45 minutes. Interviews were conducted over approximately two months, with up to four interviews being conducted in one day. After the first two interviews, we consulted with a qualitative research methodology committee that had not been involved in developing the research objectives. The group included a mix of clinicians and non-clinicians who reviewed the interview process to ensure the quality of the questions and consistency of the process. To increase internal validity of the study, at the end of each interview day, the interviewer wrote memos to describe the interview context, document emerging themes, and to review the interview process. By writing memos, we were able to identify and protect against potential biases in and preserve consistency of the interviews. Finally, we continued recruitment and interviews until we reached saturation. Saturation was judged by the authors with feedback from the external qualitative research methodology committee [30].

### Analysis

We chose an open-coding analytic approach to maximally capture physician attitudes and behaviors. Two authors [CAH and HQH] independently coded the data to identify emerging themes on an ongoing basis during the data collection. The analysts used Nvivo 9 software (QSR International, Inc.) to store, organize, and iteratively refine emergent themes in the data [31]. After coding each interview, the analysts wrote reflective memos to describe emerging themes and relationships between themes. The memos helped ensure consistency in the analytic process and to reduce the risk of introducing individual biases. Throughout the process, each analyst periodically reviewed, merged, and hierarchically organized their themes as they uncovered similarities and relationships between themes. Next, the analysts merged their independently derived codes and arrived at a final set of themes via consensus. Finally, our team, including a pain psychologist, general internist, and pain medicine specialist reviewed the emergent themes and assessed their face and content validity.

## Results

Our final sample included physicians from one private, one U.S. Veteran’s Health Administration (VA), and seven academic-affiliated practices (Table 1). The sample included nine family medicine and six general internal medicine physicians and seven men and eight women.

**Table 1:** **Physician participant characteristics**

**Physician #**	**Sex**	**Specialty**	**Years in practice**
1	Female	Internal Medicine	21
2	Female	Internal Medicine	8
3	Female	Family Medicine	9
4	Male	Family Medicine	4
5	Female	Family Medicine	7
6	Male	Internal Medicine	3
7	Female	Internal Medicine	14
8	Male	Family Medicine	25
9	Male	Internal Medicine	7
10	Male	Internal Medicine	32
11	Male	Family Medicine	11
12	Male	Family Medicine	22
13	Female	Family Medicine	25
14	Female	Family Medicine	18
15	Female	Family Medicine	6

*Note: Physician # indicates the order in which the interviews were conducted.*

### Physicians’ information needs and use

Physicians discussed the types of information that are important to them during clinical encounters for chronic noncancer pain and how that information affects their prescribing decisions.

#### Importance of objective and consistent information

Physicians frequently described their desire for objective and consistent information about patients’ pain, such as imaging that shows musculoskeletal pathology. One physician said: “*I also look to see if he’s got legitimate reasons for pain, ‘cause sometimes patients’ pain is way out of proportion to objective findings, like radiographic findings”* (Physician 2). Along the same lines, physicians reported skepticism about the value of patient self-reported pain (i.e., 0-10 numeric rating scale). Physicians expressed concern about the inconsistency of self-reported pain, observed pain behaviors, and exam findings. For example: *“Somebody will say, ‘Oh, I have ten out of [ten] pain”, but they’re sitting there, comfortably. They’re walking around, totally fine, and you’re like, ‘This is not what I would consider ten out of ten pain”,* (Physician 15). Similarly, another physician expressed frustration about the legitimacy of pain presentations that are inconsistent with physical exam:

*A lot of people come in with this chronic back pain that—it should present one way but they’re in pain all over the place. Their pain is just completely ridiculous, out of proportion to exam. You just barely touch them. ‘Ahhh!’ Jumping off the table—you know it’s like, all right, it’s probably not real* (Physician 3).

As a consequence of their challenges to assess pain, physicians frequently described concern about the appropriateness of prescribing opioids.

#### Importance of identifying “red flags” related to risks of prescribing opioids

Numerous physicians mentioned the importance of identifying “red flags” that might indicate a patient is medication seeking, at risk of addiction, or otherwise not a good candidate for opioids. Red flags emerge from different information, including inconsistencies between subjective and objective assessments:

*I do get nervous if their pain is way out of proportion to objective findings. We do get outside records. If they saying that they have very mild degenerate disc, but there’s 10 out of 10 [pain score] and wanting opiates, that gives me red flags too. Those are the hardest ones for us because they’re subjectively in so much pain but objectively they’re not* (Physician 2).

Other potential red flag information that physicians seek and can trigger concerns about opioid appropriateness included new patients, patients traveling from another county, patients who are unwilling to try non-opioid therapies, patients who request opioid dose increases, psychiatric history, drug or alcohol abuse history, suspect prescription refill history, and unsolicited patient reporting of pain scores. For example, when asked if he assesses patients using standardized pain scales, one physician said:

*Generally not, scale of one to ten, I do not. That’s a red flag. Get a new patient and they walk in and, ‘Doc, my back really hurts, and it’s a nine or ten out of ten.’ [Laughter] Where did they learn that?* (Physician 11).

#### Importance of information about physical function and outcome goals

Multiple physicians described the importance of patients’ physical function as the most important outcome. At times, physicians described their value of physical function in the same context as their devaluing of pain scores as subjective and less important outcomes. One physician explained it succinctly:

*I have this little speech I do with everyone that says, ‘The goal of this medicine is not to get rid of all your pain because if you’re looking for that, I can’t do that for you. But I can, hopefully, make you more functional.*’ *That’s the approach I use* (Physician 8).

Another physician went further and described the important of individualized approaches to identifying and assessing patients’ functional goals:

*Their function might be tailored to, and it probably should be directed at trying to what would motivate them to get going. Be it maybe playing with their grandkids, or walking with their wife, or, so I think the functional thing should be something. This never happened, but it should be discussed at that visit before the opioids are started. What are we trying to accomplish here?* (Physician 10)

Similarly, other physicians described how setting goals and expectations that are important to the patient can help them confidently prescribe opioids as well as taper or discontinue them if patients’ explicit goals, such as weight loss or function, are not met.

#### Importance of tacit knowledge and trust in patients

Some physicians went beyond clinical findings and outcome measures when discussing the information they use. They described their reliance on harder-to-measure information, such as their history with and “sense” of their patients’ need for opioids. One physician explained “*I’ve known some of my patients for over 20 years. Whether it’s right or wrong, I guess I have a sense of whether they’re someone that I feel could or would appropriately use these medications*” (Physician 8). These reports suggested that some physicians go beyond explicit, objective assessments and use more intuitive judgments and decisions about appropriateness of opioids. Multiple physicians’ described how their impressions of patients’ trustworthiness, based on their accumulated experience over time, increased their willingness to prescribe opioids. In contrast, some physicians described their relative unwillingness to prescribe opioids to patients for whom they did not have a long-standing relationship. One physician explained how he trades off his need for specific information to “check” on patients’ opioid-related risks based on his accumulated trust:

*So for people that I don’t trust, I use that outside information to evaluate whether or not this should be continued in an ongoing relationship or not. The more I trust them, based on how much I know them, the less likely I am to sort of do drug tests on them or do kind of checks on them in some way* (Physician 6).

The same physician described how for some of his most trusted patients who are taking chronic opioids, he formally documents his trust in their medical record, so that they won’t be judged negatively by other clinicians:

*With me, because I know so many details about them and I know their kids and know what’s going on in their life. I know their day-to-day affairs, I’m not worried like they [other clinicians] are when they go into the hospital and they only see the medication list. I had a patient where that was the case here recently. So I kind of documented some of my trust and my history with that patient on the chart when they went into the hospital …. (Physician 6)*

### Other decision making challenges related to opioids

Physicians described a number of other serious decision making challenges associated with prescribing opioids in the context of chronic noncancer pain.

#### Weighing potential therapeutic benefits against opioid risks

Physicians reported struggles in weighing potential therapeutic benefits of opioids versus risks, such as the risks that patients are being dishonest about their pain and/or are at risk of abusing, misusing, or diverting opioids. Some physicians described risks of side effects and adverse events, but physicians more often emphasized risk of misuse, abuse, and diversion. One physician categorized his patients, saying:

*You have the patients who definitely need the medications. You have the patients who definitely don’t need the medications. Then you have the really tough management cases of the ones in between that are difficult to assess whether or not they truly do need the medication (Physician 12).*

The same physician admitted that despite his confidence, he knows he has misjudged patients’:

*I would say the vast majority of people I think I get a good feel for it. But I’ve been snagged so many times [laughs]. I could have sworn this person was being straight with me* [in reporting their pain]*. … I would imagine you’re not 100 percent right all the time. … So sometimes you’re probably erring in … not giving somebody pain medications when they truly do need it. And you’re gonna err sometimes in giving patients medications when they don’t need it. (Physician 12)*

Another physician described a patient who received opioids and was using cocaine:

*I did a couple of urine screens and things like that. I was a little uncomfortable, but she was a patient for a long time. It seemed to keep her out of the emergency room. If I didn’t give her pain medicine, she’d end up in the emergency room. Anyway, just recently I found out that she came into the hospital and she was positive for cocaine a couple of times. I was like, oh. You know, sometimes I don’t know how to manage them effectively ….* (Physician 1)

In addition to wanting to keep the patient out of the emergency room, this physician suggested she prescribed opioids because of her long-term relationship and desire to trust the patient, saying: “*I mean she's been my patient a really long time. We've kind of been through a lot of things together* (Physician 1).

In contrast, another physician said her struggles to judge opioid risks have led her to avoid prescribing opioids altogether:

*There’s this sense of, you feel like you’ve been taken advantage of, and you were out there trying to help them, and instead, they’re abusing the medication. In a way, you’re either harming them or harming someone else out in the community, and so, there is that feeling of, you don’t wanna be one of those docs. … It’s kind of like, you get bit several times, and then, you’re like, ‘I’m just not gonna give pain meds to anybody.’* (Physician 15)

#### Time and resource constraints

Physicians also said that time and other resource constraints impede their ability to manage chronic opioids effectively. For example, one physician said:

*The university has increased the number of patients you are required to see ever since I’ve been here. … It’s racing from one patient to another, and sitting down and doing formal assessments is hard to work in. … but formal assessment scales and these lengthy conversations that you would think family practitioners would sit down and spend all this time, this practice isn’t set up to do that. It’s set up to move 35,000 patient visits through a year* (Physician 11).

Similarly, a VA physician described how managing other chronic conditions can crowd out time to effectively manage pain and opioids: *“you’re given a 30-minute appointment at the VA, and you’ve got a lot to document, and you got to see about their diabetes and their hypertension, and their lipids, and all this other stuff. Pain really never, it’s sort of a labor intensive thing treating pain, so it takes time”* (Physician 10). In addition to time, some physicians perceived a need for resources such as secure urine drug screening processes and access to other specialists, saying, for example:

*I find that hard because our office isn’t really set up for that [urine drug screens]. I really don’t know about the security. … Occasionally, I’ll do a random one, but I know I should do them more, but I don’t. It’s just too complicated. … They’ve [patients who have gone to pain specialty clinics] described to me the process; that there’s someone monitoring their urine when they come out of the bathroom or something. That’s not true of our clinic, we’re not set up that way. Then I think, well, should I even bother?* (Physician 1)

#### The role of primary care specialties in managing pain

Physicians offered multiple perspectives on their preparedness to manage chronic opioid therapy and on the role of primary care in chronic pain. For example, a family and an internal medicine physician each described how, over time, they began to specialize in pain care. As one said:

*Because the thing is we are—family is so broad that people can specialize in all type of stuff and still be called a family physician. It’s accordin' to what your interests lie. My interests started with chronic pain management because of my previous jobs (Physician 14).*

She further described how an increasing volume of patients with chronic pain led her to enroll in pain-related continuing education to learn about alternatives to opioids: “*I felt like, ”okay, this cannot be good medicine. I’m sittin’ here writin’ 20 pills. I have got to find a better way to do this.“ At that point that’s when now all my CMEs are goin’ this way*” (*Physician 14).* Similarly, one of the internal medicine physicians from the VA described how his volume of patients on chronic opioids led him to “specialize” in pain:

*It’s [pain] been, it’s become an interest of mine in the last four or five years. I did none in private practice. Then when I came back here [the VA] and inherited a practice with just, probably, I think as much as 20 percent of primary care is on opioids, so you come into this system and all of a sudden you see all these people on high dose, long acting medicines with very little risk assessment, very little monitoring, very little safety stuff that you normally have in place to make sure the drugs were being used safely. (*Physician 10)

In contrast, other physicians described how they actively avoid patients with complex chronic pain conditions and/or on chronic opioids therapy. For example, one said: “*I guess when you’re in primary care you can choose. It’s not like you’re specializing but some of the docs decide to go ahead and do more pain management so I guess I didn’t go that route”* (Physician 5). Altogether, physicians described varying levels of interest in caring for chronic pain and willingness to prescribe chronic opioids.

## Discussion

Physicians in our U.S.-based study described a range of decision making challenges, including uncertainty in diagnosing chronic non-cancer pain conditions, discomfort assessing opioid-related risks and benefits, a lack of time and resources to provide pain care, and trouble establishing trust in patients. As a product of these challenges, our study indicates that some primary care physicians actively avoid caring for chronic noncancer pain. Given the enormous prevalence of chronic pain relative to pain specialist physicians in the U.S. [1], this finding raises serious concerns about patients’ access to effective chronic noncancer pain care.

Our study also provides insight into how some primary care physicians’ judgments are likely molded by perceptions of insufficient training, misinformation, conflicting information, high uncertainty, frustration, and mistrust when managing chronic opioids for pain. Others have described such clinical scenarios as “information chaos,” which may harm physician performance and patient safety [32]. Thus, our findings suggest the potential value of electronic health record (EHR)-based decision support that fosters clinical team-based approaches to information management [32] that ensure physicians are able to diagnosis pain conditions, weigh opioid risks and benefits, and judge patient trustworthiness more objectively. For example, decision support that collects and communicates individualized information on the etiology and classification of patients’ pain or on medication options could help physicians more confidently and accurately deliver evidence-based care. Similarly, decision support could automatically calculate patients’ risk of opioid abuse and display results to a physician in the context of opioids’ expected benefit relative to other treatments. However, such efforts to improve physicians’ decisions must be based on established medical evidence and carefully integrated into clinical interactions so not to adversely affect workflows or physician-patient communication and trust [33,34]. Indeed, a prior attempt to implement guideline-based decision support for chronic opioid therapy was harmed by clinician time constraints, competing priorities, and discomfort communicating with patients about opioids [15].

To complement decision support, our study also suggests the need for education and policies that support high quality primary care for chronic noncancer pain. Like prior work in an inpatient setting [35], our study clearly showed that some physicians mistrust their patients’ self-reported pain and desire objective measures, such as imaging results indicating musculoskeletal pathology. But, this may reflect a basic lack of understanding that many pain conditions, such as centralized pain, cannot be assessed with routine physical exam or standard X-Ray, MRI, or CT imaging techniques [36]. Consistent with the literature ([37,38], we also found that physicians often rely on tacit assessments of patients’ risk of aberrant drug-related behaviors, such as opioid abuse, despite recognizing they may make inaccurate assessments. Therefore, clinical leaders, educators and policymakers must continue to create and disseminate usable, evidence-based education on chronic pain and opioid risk assessment. In particular, educators should aim to correct judgments that “legitimate” pain is always observable in objective information, that “pain care” is equivalent to prescribing opioids, or that risk of aberrant drug-related behaviors is easily intuited. In broad terms, it must be acknowledge that patients with chronic pain conditions can be time-consuming and emotionally tiring to treat [39], but pain, like diabetes or hypertension, can be effectively treated in primary care settings [40].

This study has some limitations. First, we aimed to obtain rich information from a small, diverse sample of physicians in one U.S. state. As a consequence, our results do not allow us to make general or representative conclusions about physician populations. However, our sampling strategy was successful in recruiting physicians who varied in terms of age, gender, practice location, practice ownership, and experience and training in pain care. Yet, it is possible that our sampling did not capture some physician decision making approaches, such as those that vary across cultures or larger international boundaries. Therefore, we would be cautious in ascribing transferability of our results to international settings. Another limitation is the potential that our results reflect predispositions or other biases of the data analysts rather than purely reflecting the phenomenon being studied. To mitigate these risks, we used multiple data analysts. And, each analyst wrote reflective memos during the analysis to help verify their processes and ensure that emergent themes reflected the study data rather than internal biases. Finally, our data reflect physicians’ perceptions of how they make decisions, which may reflect their sensemaking process, social desirability, or actual decision making [41-43]. While we did not observe actual physician behavior, we did ask numerous follow-up questions to probe beyond potentially rehearsed responses. Encouragingly, physicians reported a range of decision making strategies and admitted that their decisions sometimes led to poor outcomes. Therefore, we believe that our results are reasonably reflective of actual decision making processes.

## Conclusions

This study contributes new insights into decision making challenges that primary care physicians face in managing the many patients with chronic noncancer pain. In particular, we identified and richly described physicians’ struggles to deliver high quality care as they seek and make decisions based on an array of incomplete, conflicting, and often untrusted information about their patients. Future work should develop and disseminate decision support and education that corrects misconceptions about chronic pain assessment and opioid management while also providing usable point-of-care information that meaningfully aids physician-patient interactions.
